# Effects on Lipid Profile after One Year of Apremilast Therapy in Patients with Psoriasis: A Monocentric Experience

**DOI:** 10.3390/life14030395

**Published:** 2024-03-16

**Authors:** Prisca Guerra, Antonella Di Cesare, Elia Rosi, Ilaria Scandagli, Gianmarco Silvi, Giulia Nunziati, Francesca Prignano

**Affiliations:** Department of Health Sciences, Section of Dermatology, University of Florence, 50125 Florence, Italy

**Keywords:** psoriasis, psoriatic arthritis, apremilast, comorbidities, metabolic effect, lipid profile, weight, cholesterol, HDL, triglycerides

## Abstract

Apremilast, a phosphodiesterase-4 inhibitor, has shown promise to have a potential beneficial metabolic effect. We conducted a single-centre retrospective study on adult patients with moderate-to-severe psoriasis who underwent apremilast treatment over at least 12 and 52 weeks, respectively. Baseline characteristics, weight, lipid profile, and fasting glucose levels were collected at baseline and at 12, 24, and 52 weeks. Furthermore, we conducted a narrative review of the current scientific knowledge on the metabolic effects of apremilast in patients with psoriasis and psoriatic arthritis. We observed a significant reduction in average weight and body mass index (BMI) in patients treated with apremilast in both the initial and the subgroup analysis, a significant reduction in triglycerides levels at 24 and 52 weeks, and a significant increase in high-density lipoprotein (HDL) levels at 52 weeks, whereas there were no significant changes in total cholesterol or low-density lipoprotein (LDL) concentrations over the 52-week treatment period. These findings suggest a potential positive impact of apremilast on both weight management and lipid profile in individuals with moderate-to-severe psoriasis in the medium–long term.

## 1. Introduction

Psoriasis, traditionally regarded as a chronic immune-mediated disease affecting primarily skin, is currently recognized as a multisystemic disease with implications beyond the skin. Patients with psoriasis exhibit a significantly elevated risk of cardiovascular and metabolic comorbidities, including hypertension, type 2 diabetes mellitus (DM2), obesity, metabolic syndrome (MetS), and non-alcoholic fatty liver disease (NAFLD) [1]. The association with these comorbidities is likely the result of chronic and systemic inflammatory processes shared with psoriasis [2]. Therefore, a comprehensive approach for patients with psoriasis is crucial, not only to improve their quality of life but also to prevent and manage these significant comorbidities. 

In recent decades, several novel treatments have been approved for the treatment of psoriasis, including biologics and small molecules. Among the latter, apremilast, a phosphodiesterase-4 inhibitor (PDE4i) approved by the EMA and the FDA for the treatment of moderate-to-severe psoriasis and psoriatic arthritis (PsA), has been suggested by many clinical studies to have a potential beneficial metabolic effect.

In view of these considerations, a single-centre retrospective study was conducted among patients attending the psoriasis outpatient clinic of the University of Florence in order to explore the effects of apremilast on weight and lipid profile after 12 weeks and 52 weeks of therapy in patients with a diagnosis of moderate-to-severe psoriasis.

Furthermore, we present a narrative review of the current scientific knowledge on the metabolic effects of apremilast in patients with psoriasis and psoriatic arthritis.

## 2. Materials and Methods

Adult patients with a confirmed diagnosis of moderate-to-severe psoriasis who underwent apremilast treatment for over 12 weeks attending the psoriasis outpatient clinic of the University of Florence were included in the first analysis of our study. A sub-group analysis was then conducted in those patients treated with apremilast for at least 52 weeks. Medical records of eligible patients were retrospectively reviewed. Baseline characteristics, including age, gender, psoriasis severity assessed trough psoriasis area severity index (PASI), comorbid PsA, body mass index (BMI), smoking habit, relevant medical history, previous psoriasis systemic and biologic treatments, and concomitant therapies were collected from patient’s records. Weight measurements and lipid profile parameters, [i.e., total cholesterol, low-density lipoprotein (LDL), high-density lipoprotein (HDL), triglycerides], and fasting glucose levels, all routinely assessed in the outpatient visits, were collected from record of evaluations at baseline (T0) and at 12, 24, and 52 weeks. Patients with incomplete biochemistry data were excluded from analysis.

Descriptive statistics were used to summarize baseline features, while a paired parametric *t*-test (PRISM version 10.2.0) was employed to analyse changes in weight and lipid profile parameters over the 12- and 52-week period. Spearman’s rank correlation test was used to quantify the association between baseline PASI and baseline weight, BMI and lipid blood levels and to quantify the association between weight and lipid level variations compared to PASI changes at 52 weeks. The significance level was set at *p* < 0.05. 

A comprehensive literature search in the electronic database PubMed was conducted up to 31 December 2023, using the search terms “psoriasis” OR “psoriatic arthritis” in combination with “apremilast” AND “weight” OR “BMI” OR “lipid profile” OR “metabolic” OR “glycaemic” OR “cardiovascular”, to identify relevant English-language publications. All articles were screened on the base of the content of the title and abstract to select the studies on metabolic effects of apremilast in psoriasis or PsA, formulating a hypothesis about its influence on weight, lipid profile, glucose homeostasis, and cardiovascular risk in these diseases. References of relevant articles were also manually searched for possible inclusion in the present review. As a result of the methodological criteria applied in this review, there was a risk to exclude relevant articles based on not satisfying any of these criteria. This could have inadvertently resulted in the omission of studies investigating PDE4 inhibitors more broadly, potentially providing additional insights into the topic.

## 3. Results

**Figure 1:**
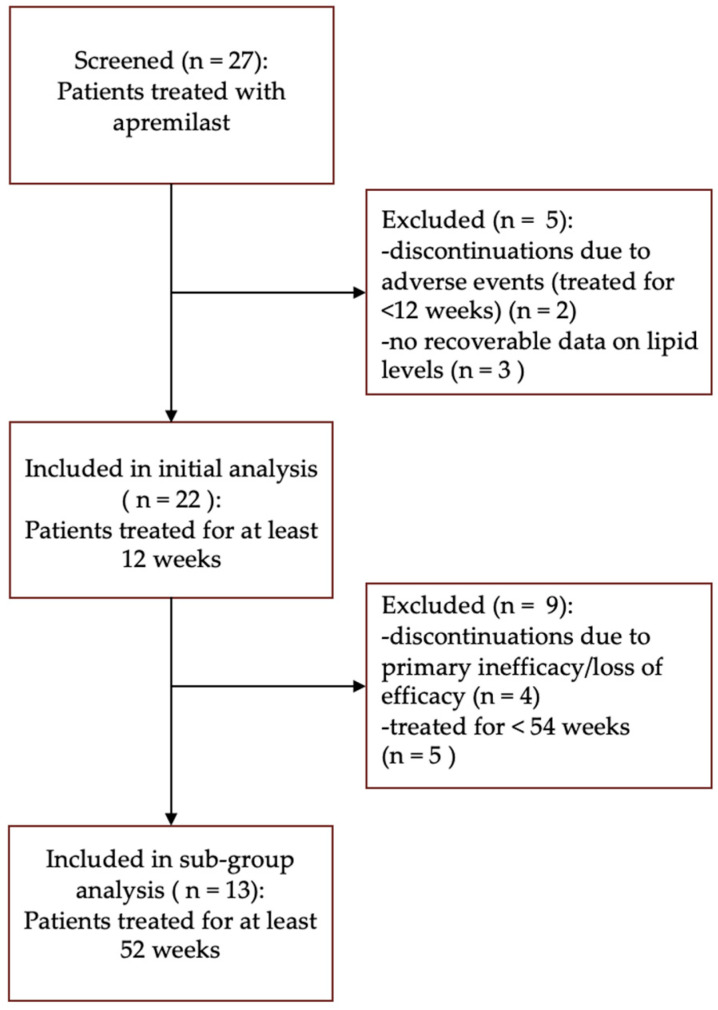
Patients’ selection criteria.

A total of 27 patients with psoriasis treated with apremilast were screened. Of the 27 patients, 2 patients discontinued apremilast before week 12 due to adverse events (gastrointestinal symptoms and COVID-19-related fatality). Additionally, three patients were excluded from analysis due to missing laboratory assessments of serum lipid levels. Data from the remaining 22 patients, who completed at least 12 weeks of therapy, were included in the initial analysis. Among these patients, five received treatment for less than 52 weeks, while four discontinued apremilast due to primary inefficacy or loss of efficacy before the 52-week endpoint. Subsequently, 13 patients met the criteria for inclusion in the subgroup analysis, having undergone treatment for at least 52 weeks (Figure 1).

### 3.1. Population Characteristics at Baseline and Initial Analysis

Patients’ baseline characteristics are shown in Table 1. The initial analysis showed a significant reduction in body weight and BMI at 12 weeks of treatment (*p* < 0.001), with a mean difference of −1.86 kg [95% Confidence interval (CI) −2.7; −0.98] at 12 weeks. Regarding lipid profile parameters, there were no significant changes in total cholesterol, LDL, HDL, or triglycerides concentration over the 12-week treatment period [Table 2].

### 3.2. Sub-Group Study Population Characteristics at Baseline

The average age of the patients was 63.8 ± 10.0 years. Out of 13 patients, 3 were male (23.1%) while 10 were female (76.9%). Four out of thirteen patients (30.8%) had a concomitant diagnosis of PsA. The mean disease duration was 22.5 ± 12.6 years. The average BMI of the patients was 26.3 ± 3.4 and the average baseline PASI score of the patients was 7.5 ± 3.6. All patients (n = 13) had at least one comorbidity, in particular hypertension (n = 8, 61.5%), DM2 (n = 2, 15.4%), hypercholesterolemia (n = 6, 46.2%), a personal history of malignancy (n = 4, 30.8%), obesity (defined as BMI ≥ 30) (n = 1, 7.7%), an occult/latent infection (in particular hepatitis B virus and latent tubercular infection) (n = 8, 61.5%), and previous transplant (n = 1, 7.7%), among other comorbidities. Eleven out of thirteen patients were undergoing concomitant therapies; notably, eight (61.5%) patients were taking antihypertensive drugs and four (30.8%) patients were taking statins. None of the patients were taking either fenofibrates or omega-3 fatty acids for treatment. Out of 13 patients, 53.8% (n = 7) patients were previously treated with methotrexate, 15.4% (n = 2) with cyclosporin, and 7.7% (n = 1) with acitretin. Twenty-three % (n = 3) were naïve to systemic treatment and 61.5% (n = 8) of patients were bio-naïve, while 38.5% (n = 5) of patients were previously treated with anti-TNF alpha molecules and one (7.7%) patient had been previously treated with secukinumab [Table 3]. No significant correlation between baseline BMI and lipid blood levels with baseline PASI was observed.

### 3.3. Weight and Lipid Parameters

We observed a significant reduction in body weight at 12 (*p* < 0.001), 24 (*p* = 0.001) and 52 weeks (*p* = 0.021) of treatment, with a mean difference of −2.7 (95% CI −4.9; −0.5) kg at 52 weeks. Similarly, there was a significant reduction in BMI at 12 (*p* < 0.001), 24 (*p* = 0.001) and 52 weeks (*p* = 0.018) of treatment. No significant correlation between weight loss and PASI change was demonstrated at 52 weeks. Regarding lipid profile parameters, the study population showed a significant reduction in triglycerides levels at 24 (*p* = 0.028) and 52 weeks (*p* = 0.013), with a mean reduction of 23.3 mg/dl (18.21%) (95% CI −40.8; −5.8) at week 52. Patients with higher baseline triglycerides levels were those with greater reductions in triglycerides values at 52 weeks. Notably, none of the study participants implemented therapy with lipid-lowering drugs (e.g., fenofibrates or omega-3 fatty acids) during the 52 weeks. Moreover, a significant increase in HDL levels was observed at 52 weeks (*p* = 0.015). Interestingly, the triglycerides/HDL ratio significantly (*p* = 0.01) decreased over the 52 weeks of treatment. There were no significant changes in total cholesterol nor LDL concentration over the 52-week treatment period, but we observed a significant (*p* = 0.003) reduction in total cholesterol/HDL ratio at week 52. No significant correlation between changes in triglycerides and HDL blood serum levels and PASI variation was observed at 52 weeks. Furthermore, we did not observe a significant variation in fasting glucose serum levels [Table 4]. All significant data were confirmed in multivariate analysis (RM 1-way ANOVA with Tukey’s multiple comparisons test). 

## 4. Discussion

In the current study, we observed a significant reduction in average weight and BMI in patients treated with apremilast in both the initial and the subgroup analysis. Notably, weight loss predominantly occurred during the first 12 weeks of apremilast treatment and gradually decreased during treatment continuation. Interestingly, the observed weight loss was greater in overweight patients and led to a normalization of the average weight among patients, indicating a positive trend in overweight individuals without posing a threat to their health. Nonetheless, as weight loss is still reported as a side effect of apremilast [3], and substantial weight loss may not necessarily be beneficial, it should be carefully monitored, especially in underweight patients. In the subgroup analysis, we furthermore observed a significant decrease in triglycerides levels, alongside an increase in average HDL levels over 52 weeks of apremilast therapy. Remarkably, the improvement in serum lipid levels was significant only after 24 and 52 weeks for triglycerides and HDL, respectively, rather than in the short term. Indeed at 12 weeks, although not significant, there was a slight increase in triglycerides values in both the initial and the subgroup analysis. Interestingly, we did not observe a significant correlation between PASI improvements and changes in BMI or HDL or triglycerides levels. This might be explained, on the one hand, by the small sample size of patients in the study and, on the other hand, by the relatively low PASI of the patients at baseline, as almost all patients had received previous systemic and/or biologic treatment. These findings underscore the potential beneficial impact of apremilast in the medium–long term on both weight and lipid profiles in individuals with moderate-to-severe psoriasis within a clinical setting. An increase in triglycerides levels and a decrease in HDL levels have been associated with MetS and cardiovascular diseases (CVDs), and their ratio, triglycerides/HDL, has been proposed as a novel biomarker for predicting the risk of both clinical entities. However, there is a lack of consensus on the universal cut-off value of the triglycerides/HDL ratio to be used as a predictive biomarker [4]. Indeed, despite meeting targets for LDL cholesterol, blood pressure, and glycemia, patients with dyslipidaemia are still exposed to high residual cardiovascular risk. Elevated triglycerides and low HDL cholesterol contribute significantly to this risk [5]. Therefore, even though our study did not directly assess the effect of apremilast therapy in psoriatic patients on cardiometabolic risk, the observed reduction in triglycerides and the increase in HDL levels, and consequently an improved triglycerides/HDL ratio, aligns with the broader goal of CVD and MetS risk reduction in psoriatic patients and further supports the potential cardiovascular benefits of apremilast in psoriasis management.

Additionally, the real-life setting of this research highlights the complex nature of psoriasis management in a population with multiple comorbidities, emphasizing the importance of therapeutic strategies that address both dermatological and systemic health concerns. Within the complexities of real-world patient care, apremilast emerges not only as an effective treatment option for psoriasis and psoriatic arthritis but also as a potential ally in managing metabolic comorbidities and mitigating CVD risk in these patients. 

The findings presented herein significantly contribute to our understanding of the proper treatment choice of apremilast therapy in patients with cardiometabolic disease with either severe or moderate psoriasis. Recent investigations have highlighted the potential beneficial effects of apremilast treatment on various aspects of metabolic health. Specifically, studies have explored the impact of apremilast and PDE4 inhibition on weight loss, adiposity distribution, lipid parameters, glucose homeostasis, and cardiovascular risk factors. Herein, we present a narrative review of the existing knowledge in these areas.

### 4.1. Lipid Parameters

To the best of our knowledge, there is little literature available on the effects of apremilast therapy on serum lipid values. In a case report of a PsA patient treated with apremilast, notable improvements in the serum lipid profile were observed. After 4 weeks, there was a 5.8% reduction in total cholesterol and a decrease in the total cholesterol/HDL ratio. Subsequently, at the 12-month mark, there was a further reduction in total cholesterol, LDL, and triglyceride values, amounting to 15.6%, 25.7%, and 17.7%, respectively. Additionally, the patient presented a 20% increase in HDL levels compared to baseline values [6]. In a 6-month prospective open-label study involving patients with PsA and psoriasis receiving apremilast, an initial modest reduction in total cholesterol, HDL, and LDL concentrations was reported with treatment. However, these values returned within baseline after 6 months. Furthermore, no significant change in triglycerides concentration was observed [7]. The discrepancy of results with our study might be attributed to differences in both the study population and design. Our study included a higher percentage of individuals with dyslipidaemia compared to the one by Ferguson et al. Additionally, the duration of observation in our study spanned a longer period (52 weeks versus 6 months), potentially allowing for a more comprehensive assessment of changes in lipid parameter values. Notably, in another study, lower cholesterol LDL levels at baseline were observed to represent a favourable response factor of apremilast treatment [8].

### 4.2. Weight Loss and Adiposity

Consistent with the findings of our investigation, registries studies have reported weight loss as a noticeable side effect in patients with PsA or psoriasis treated with apremilast [9,10,11]. A pooled analysis of the ESTEEM 1 and ESTEEM 2 phase 3 trials revealed a mean percent change from baseline weight loss of 1.53 over the 156 weeks of apremilast treatment. Notably, the proportion of patients reporting weight loss appeared to be higher among those with a higher baseline BMI, and the weight loss predominantly occurred in the first year of apremilast treatment. Furthermore, no association was found between gastrointestinal adverse events, such as diarrhoea, nausea, or vomiting, and weight loss in apremilast-treated patients [12]. Various reports in the existing literature also highlight a reduction in weight and/or BMI during treatment with apremilast [13,14]. In a 6-month prospective open-label study involving patients with PsA and psoriasis receiving apremilast, a consistent reduction in weight and BMI was observed across all time points compared with baseline. The study revealed a mean weight loss of 2.2 kg, a significant decrease in waist circumference at month 1, and a reduction in hip circumference at month 3. However, there was no overall change in the waist-to-hip ratio by the study’s conclusion. Additionally, a reduction in subcutaneous fat, but not visceral fat, was detected through magnetic resonance imaging (MRI) [7]. Conversely, in a nonrandomized clinical trial involving psoriatic patients treated with apremilast, a 5% to 6% decrease in both subcutaneous and visceral fat was established. This reduction became evident at the 16-week mark and remained sustained throughout the 52-week treatment period [15]. Indeed, the differentiation between visceral and subcutaneous fat has become crucial in assessing this CVD risk. Visceral fat is known for its metabolic activity, produces peptides, and influences cardiovascular homeostasis. Dysregulation of visceral adipose tissue inflammation alters immune cell and adipokine profiles, exacerbating endothelial dysfunction [16,17]. Therefore, the observed reduction in both subcutaneous and visceral fat in patients treated with apremilast suggests a potential protective effect against CVD risk factors. Although current data on the efficacy of apremilast in these aspects are limited and further investigations are warranted, the reduction in visceral fat in patients treated with apremilast suggests an additional potential protective effect against CVD risk factors.

Furthermore, weight loss intervention has been correlated with a reduction in the severity of psoriasis in overweight or obese patients [18] and, beyond being a well-established risk factor for CVD, obesity and high BMI have been linked to a reduced short-term clinical response to various systemic treatments, specifically to biologics [19]. Hence, the observed effects of apremilast on weight loss and adiposity reduction may not only contribute to improved metabolic profiles but also enhance the clinical response to treatment in this patient population.

### 4.3. Glucose Homeostasis

In a 52-week open-label trial on a cohort of psoriatic and PsA patients treated with apremilast, Mazzilli et al. reported a reduction in fasting glucose levels over time [8]. These results are in contrast with those of Ferguson et al., who did not observe any significant improvement in glycaemic status and GLP-1 levels after 6 months of treatment with apremilast. Nonetheless, the researchers hypothesised that the discrepancy in results might relate to the differences in study population, as the first study included a significantly greater proportion of individuals with diabetes than the latter [7]. Indeed, in a post hoc analysis of pooled data from 1808 participants from the ESTEEM, LIBERATE, and PALACE trials, a reduction in glycated haemoglobin (HbA1c) levels was observed in patients with an elevated baseline HbA1c (≥6.5%) [20]. Similarly, in a cohort study on 45 biologically naïve patients treated for 6 months with apremilast, MTX, or combined therapy, the homeostatic model assessment insulin resistance (HOMA-IR) levels were significantly reduced after 6 months of therapy in patients with high baseline HOMA-IR levels in the apremilast group, whereas HOMA-IR levels in patients with low baseline HOMA-IR levels remained unaffected after any of the three therapeutic strategies [13]. Indeed, various studies suggest that PDE4 plays an essential role in glucose metabolism and therefore PDE4 inhibition might exert a positive effect on glucose homoeostasis. Roflumilast, a PDE4 inhibitor, demonstrated a modest induction of glucose-dependent insulin release in vitro [21], a delayed progression of diabetes in diabetic mice models via improvement of glycaemic variables and protection of pancreatic islets [22], and a glucose-lowering effect in patients with newly diagnosed DM2 [23]. Moreover, rolipram, another PDE4 inhibitor, exhibited increased secretion of glucagon-like peptide-1 (GLP-1) in intestinal GLUTag cells and non-diabetic rats [24]. In our small cohort of patients, apremilast seemed to exert a neutral effect on fasting glucose levels; however, data on HbA1c were available from only few patients at all timepoints, so our results do not add relevant clinical information to this topic. 

### 4.4. Cardiovascular Effects

In a 2021 trial, Ikonomidis et al. compared the effect of apremilast, cyclosporin, and etanercept on CVD risk factors such as endothelial glycocalyx integrity and vascular and left ventricular myocardial function in psoriasis. After 4 months, the researchers observed a restored glycocalyx integrity in patients treated with apremilast, along with a greater improvement in vascular and myocardial function compared to those treated with etanercept or cyclosporine [25]. On the other hand, Ferguson et al. observed no improvement in vascular function, assessed through systolic and diastolic pressure values, the reactive hyperaemia index, and the baseline median augmentation index in patients treated with apremilast [7]. 

Similarly, in a non-randomized 52-week clinical trial on patients treated with apremilast, Gelfand et al. reported no significant changes in aortic vascular inflammation with apremilast. However, they observed an overall beneficial association with a subset of cardiometabolic biomarkers, including a reduction in IL-1b, fetuin A, valine, leucine, and isoleucine levels after 16 weeks, decreases in ferritin, cholesterol efflux capacity, β-hydroxybutyrate, acetone, and ketone bodies, and an increase in apolipoprotein A-1 levels after 52 weeks [15]. Arias de la Rosa et al. also observed that treatment with apremilast significantly reduced the levels of CVD-related markers (in particular of CVD-related proteins associated with the pathogenesis of PsA, including FABP-4, GAL-3, MMP3, and CD163), particularly in patients with a higher prevalence of cardiometabolic comorbidities [13]. Moreover, a recent study on stroke in mice models found a significant efficacy of apremilast on ischemic stroke outcomes by alleviating enhanced blood–brain barrier permeability and neuroinflammation through ROCK2 inhibition, suggesting its potential use as a therapeutic option for ischemic stroke [26].

In their in vitro study on human aortic endothelial cells, Wang et al. suggested that the favourable effects of apremilast on atherosclerosis may be explained by (i) the reduction in the expression of lectin-like oxidized-LDL receptor-1, (ii) the inhibition of pro-inflammatory cytokines, including TNF-α, IL-6, and IL-8, and (iii) decreasing monocyte adhesion to endothelial cells, which is a key factor in atherosclerosis pathogenesis [27].

The favourable effect of apremilast on cardiovascular risk factors might be further explained by the findings of a study by Otto et al. on human umbilical vein endothelial cells (HUVECs) treated with tumour necrosis factor-α (TNF-α). In this research, apremilast was shown to mediate different anti-inflammatory activities by suppressing, through TNF-α inhibition, the release of key endothelial pro-inflammatory factors. This suppression, partially through a PDE4-independent signalling pathway, includes the release of granulocyte-macrophage colony-stimulating factor, vascular cell adhesion molecule-1, and matrix metalloprotein-9 [28]. 

Nonetheless, insulin resistance has likewise been associated with a detrimental effect on endothelial glycocalyx integrity [29]; thus, the effect of apremilast on glycocalyx integrity enhancement might be explained, on the one hand, by its anti-inflammatory action and, on the other hand, by its metabolic effect. 

The extensive literature on the PDE4/cAMP/PKA pathways and in vitro/in vivo studies on other PDE4 inhibitor molecules further support the potential cardiovascular role of the PDE4 pathway, including its modulation of cardiac hypertrophy and arrhythmogenesis. Additionally, this body of research underscores the pathway’s significance in maintaining vascular homeostasis by regulating various functions of vascular smooth muscle cells, endothelial permeability, angiogenesis, monocyte/macrophage activation, and cardiomyocyte function [30].

Lastly, a low incidence of major acute cardiovascular events (MACEs) is reported in patient treated with apremilast [12]. In a cohort study of 68,678 PsA patients treated with various molecules, including apremilast, TNF-α inhibitors, IL-12/23 inhibitors, IL-17 inhibitors, and conventional treatments, the incidence rates of MACEs were the lowest for users of TNF-α inhibitors and similar for all other treatments, including apremilast [31], whereas in a more recent cohort study, the risk of MACEs was greater for PsA patients treated with either IL-12/23 and IL-17, rather than TNF inhibitors or apremilast [32]. Therefore, some authors recommend apremilast as a second-line treatment in psoriatic patients with CVD [33].

### 4.5. Potential Metabolic and Cardiovascular Mechanisms of Action of Apremilast

Apremilast inhibits PDE4, therefore increasing cyclic adenosine monophosphate (cAMP) levels and influencing pathways involved in cytokine production and gene transcription. The cAMP signalling pathway depends on the activation of protein kinase A (PKA), which allows the transcription of cAMP response element-binding protein (CREB) [34].

The molecular mechanisms underlying both weight loss and a reduction in triglycerides levels and an increase in HDL levels during apremilast therapy might be represented by an increase in lipolysis. Indeed, in adipose tissue, PDE4 inhibition induces an increase in cAMP levels that, through cAMP-PKA phosphorylation, stimulates hormone-sensitive lipase (HSL). This activation of HSL enhances lipolysis, responsible for the hydrolysis of stored triglycerides into free fatty acids (FFAs) and glycerol [34]. A potential explanation for the beneficial effects of PDE4 inhibitors on cardiovascular risk factors may involve an increase in apolipoprotein A-I (apoA-I)-mediated cholesterol efflux, which facilitates the removal of excess cholesterol from macrophage foam cells within the atherosclerotic arterial wall [35]. 

On the other hand, apremilast’s role in glucose homeostasis might be mediated by an increase in cAMP levels and a subsequent increase in PKA-mediated glucagon-like peptide-1 (GLP1), which, in turn, stimulates insulin release in pancreatic β-cells [24]. 

## 5. Limitations

The main limitations of this study include its retrospective design, the small sample size of patients, and the monocentric data collection. Due to the small sample size, we were unable to explore the impact of confounding variables, such as concomitant treatments or lifestyle changes, on the observed outcomes. Our findings should therefore be interpreted as exploratory and require validation in a larger, controlled study.

## 6. Conclusions

In the medium to long term period of treatment, patients with psoriasis undergoing apremilast therapy reported a significant decrease in average weight and triglyceride levels, together with an increase in average HDL levels. These findings suggest a potential positive impact of apremilast on both weight and lipid profile metabolism in individuals with moderate-to-severe psoriasis.

Moreover, the current literature supports the notion that apremilast treatment may offer benefits for metabolic health and cardiovascular risk factors in patients with psoriasis and PsA. Further research is needed to comprehensively understand apremilast’s mechanisms of action and clinical implications. Moreover, future studies should aim to explore and compare the cardiometabolic effects of apremilast and biologic therapies in patients with psoriasis. These studies might provide valuable insights into the optimal treatment strategies and therapeutic benefits for psoriatic patients with cardiovascular and metabolic comorbid conditions.

These research endeavours might greatly improve our understanding and assist in identifying patients who are best suited for apremilast therapy within a multidisciplinary care approach involving cardiologists and endocrinologists. As a result, this might lead to tailoring apremilast prescriptions in order to maximize the therapeutic benefits for patients.

In conclusion, the findings of this retrospective study suggest that apremilast may play a valuable role in mitigating cardiovascular risk factors in individuals with moderate-to-severe psoriasis. Further prospective, controlled trials and collaborative efforts between dermatologists, cardiologists, and endocrinologists are warranted to validate and expand upon these results, ultimately refining the therapeutic approach for this patient population.

## Figures and Tables

**Table 1:** Patients’ baseline characteristics.

Total n	22
Gender F, n (%)	15 (68.2)
Age years Mean ± SD	62.7 ± 10.3
BMI Mean ± SD	27.5 ± 5.1
PsA n (%)	6 (27.3)
Disease duration years Mean ± SD	23.0 ± 13.1
Smoking habit n (%)	5 (22.7)
PASI Mean ± SD	8.0 ± 4.4

Abbreviations: SD, standard deviation; BMI, body mass index; PsA, psoriatic arthritis; PASI, psoriasis area severity index.

**Table 2:** Lipid profile values at baseline and week 12 in the initial study population (n = 22).

	Baseline	12-Week	*p*-Value
**Weight** (Kg) Mean ± SD	75.7 ± 14.5	73.9 ± 14.0	**<0.001**
**BMI** Mean ± SD	27.5 ± 5.1	26.8 ± 4.9	**<0.001**
**Total cholesterol** (mg/dL) Mean ± SD	196.4 ± 31.1	192.1 ± 35.8	0.425
**LDL** (mg/dL) Mean ± SD	120.2 ± 23.6	118.3 ± 22.9	0.589
**HDL** (mg/dL) Mean ± SD	55.6 ± 18.6	54.2 ± 18.4	0.181
**Triglycerides** (mg/dL) Mean ± SD	118.0 ± 57.1	132.0 ± 74.7	0.208

Note: Values in bold are statistically significant (parametric paired *t*-test). Abbreviations: SD, standard deviation; LDL, low-density lipoprotein; HDL, high-density lipoprotein.

**Table 3:** Sub-group study population baseline characteristics.

Total n	13
Gender F, n (%)	10 (76.9)
Age years Mean ± SD	63.8 ± 10.0
BMI Mean ± SD	26.3 ± 3.4
PsA n (%)	4 (30.8)
Disease duration years Mean ± SD	22.5 ± 12.6
Smoking habit n (%)	3 (23.1)
PASI Mean ± SD	7.5 ± 3.6
Comorbidities	
n, (%)	13 (100)
Hypertension	8 (61.5)
DM2	2 (15.4)
Hypercholesterolemia	6 (46.2)
Malignancy	4 (30.8)
Obesity	1 (7.7)
Infectious disease	8 (61.5)
Solid organ transplant	1 (7.7)
Concomitant medications	
n (%)	11 (84.6)
Antihypertensive	8 (61.5)
Statin	4 (30.8)
Oral anti-diabetic agents	2 (15.4)
Previous DMARDs	
n (%)	10 (76.9)
Methotrexate	7 (53.8)
Cyclosporin	2 (15.4)
Acitretin	1 (7.7)
Previous biologics	
n (%)	5 (38.5)
Adalimumab	3 (23.1)
Etanercept	4 (30.8)
Certolizumab	1 (7.7)
Secukinumab	1 (7.7)

Abbreviations: SD, standard deviation; BMI, body mass index; PsA, psoriatic arthritis; PASI, psoriasis area severity index; DM2, Type 2 diabetes mellitus.

**Table 4:** Lipid profile values at baseline, 12, 24, and 52 weeks in the sub-group study population (n = 13).

	Baseline	12-Week	*p*-Value	24-Week	*p*-Value	54-Week	*p*-Value
Weight (Kg) Mean ± SD	73.4 ± 11.1	71.8 ± 10.9	**<0.001**	71.3 ± 10.9	**0.001**	70.7 ± 10.1	**0.021**
BMI Mean ± SD	26.3 ± 3.4	25.7 ± 3.3	**<0.001**	25.5 ± 3.2	**0.001**	25.3 ± 2.8	**0.018**
Total cholesterol (mg/dL) Mean ± SD	203.8 ± 22.2	200.0 ± 29.0	0.61	199 ± 24.7	0.397	204.5 ± 29.2	0.902
LDL (mg/dL) Mean ± SD	126.8 ± 15.6	122.2 ± 21.2	0.315	120.5 ± 20.5	0.13	125.2 ± 17.4	0.733
HDL (mg/dL) Mean ± SD	54.8 ± 17.2	53.7 ± 16.9	0.30	57.6 ± 19.3	0.137	60.3.4 ± 18.6	**0.015**
Triglycerides (mg/dL) Mean ± SD	127.9 ± 59.7	137.0 ± 66.4	0.367	111.2 ± 50.5	**0.028**	104.6 ± 47.4	**0.013**
Fasting glucose (mg/dL) Mean ± SD	90.3 ± 10.3	93.9 ± 9.9	ns	92.9 ± 10.4	ns	91.7 ± 10.3	ns
PASI Mean ± SD	7.5 ± 3.7	3.9 ± 2.4	**<0.001**	1.2 ± 1.0	**<0.001**	1.1 ± 0.9	**<0.001**

Note: Values in bold are statistically significant (comparisons between baseline and week 12, 24, and 52, respectively; parametric paired *t*-test). Abbreviations: SD, standard deviation; LDL, low-density lipoprotein; HDL, high-density lipoprotein; ns, not significant; PASI, psoriasis area severity index.

## Data Availability

The data presented in this study are available on request from the corresponding author.
